# Manipulating the Assembly of Au Nanoclusters for Luminescence Enhancement and Circularly Polarized Luminescence

**DOI:** 10.3390/nano12091453

**Published:** 2022-04-25

**Authors:** Chen Wang, Luyao Feng, Junxiao Liu, Jing Fu, Jinglin Shen, Wei Qi

**Affiliations:** School of Chemistry and Chemical Engineering, Qufu Normal University, Qufu 273165, China; wangchen@qfnu.edu.cn (C.W.); fengluyao@qfnu.edu.cn (L.F.); liujx@qfnu.edu.cn (J.L.); fujing@qfnu.edu.cn (J.F.)

**Keywords:** Au nanoclusters, assembly, luminescence, CPL

## Abstract

Au nanocluster (AuNCs)-based luminescent functional materials have attracted the interest of researchers owing to their small size, tractable surface modification, phosphorescence lifetime and biocompatibility. However, the poor luminescence quantum yield (QY) of AuNCs limits their practical applications. Herein, we synthesized a type of AuNCs modified by 4,6-diamino-2-mercaptopyrimidine hydrate (DPT-AuNCs). Furthermore, organic acids, i.e., citric acid (CA) and tartaric acid (TA), were chosen for co-assembly with DPT-AuNCs to produce AuNCs-based luminescent materials with enhanced emission. Firstly, it was found that CA could significantly enhance the emission of DPT−AuNCs with the formation of red emission nanofibers (QY = 17.31%), which showed a potential for usage in I^−^ detection. The *n*···π/π···π interaction between the CA and the DPT ligand was proposed as crucial for the emission. Moreover, chiral TA could not only improve the emission of DPT-AuNCs, but could also transfer its chirality to DPT-AuNCs and induce the formation of circularly polarized luminescence (CPL)-active nanofibers. It was demonstrated that the CPL signal could increase 4.6-fold in a ternary CA/TA/DPT-AuNCs co-assembly system. This work provides a convenient way to build AuNCs-based luminescent materials as probes, and opens a new avenue for building CPL-active materials by achiral NCs through a co-assembly strategy.

## 1. Introduction

Gold nanoclusters (AuNCs) have attracted an immense amount of interest from researchers due to their promising features, including excellent luminescence performance [1], large Stokes shift [2], high stability [3,4], good biocompatibility [5,6] and good phosphorescence emission [7,8]. However, compared to other luminescent materials, such as organic dyes or quantum dots (QDs), AuNCs generally show low photoluminescence (PL) quantum yields (QY). Thus, various studies have been carried out to understand and improve the luminescence properties of AuNCs, including adjusting the polarity of the solvent [9,10], ligand exchanging [11], regulating the pH [12] and adding external ions [13,14]. The self-assembly strategy has attracted especial attention because of its controllable and reversible operation without the need for complicated synthesis. In recent years, our group has attempted to apply the self-assembly approach in inducing luminescence enhancement (such as co-assembly with metal ions, amino acid molecules and surfactants) [15,16,17]. However, the development of new strategies to improve the photoluminescence QY of AuNCs is still an open subject.

When non-racemic chiral luminescence systems emit left-handed or right-handed polarized light with different intensities, it is called circularly-polarized luminescence (CPL) [18,19]. The main parameter to measure the degree of polarization of circularly-polarized luminescence is dissymmetric factor (*g_lum_*). The formula is *g_lum_* = 2 (I_L_ − I_R_)/(I_L_ + I_R_), where I_L_ and I_R_ are the intensities of left and right circularly polarized light, respectively, and the value of *g_lum_* ranges from −2 to 2 [20,21]. CPL-active materials have attracted great attention from researchers because of their potential application in 3D display [22,23], biological coding [24], asymmetric synthesis [25], optical information storage and encryption [26,27]. Circularly polarized light could also be employed to prepare chiral nanostructures. Kotov et al. synthesized chiral Au nanoparticles (NPs) by implementing left- and right-polarized photon-induced reduction of HAuCl_4_, and achieved the photon-to-matter chirality transfer [28]. This contrasts with pure organic dye molecules, which have aggregation-caused quenching (ACQ) properties. Nanosized metal NCs always show aggregation-induced emission (AIE) characteristics, which might broaden the application of CPL-active materials in the aggregation state. For example, Zang’s group synthesized a pair of chiral copper clusters (R/S-Cu_14_) modified by (R/S)-2-diphenyl-2-hydroxylmethylpyrrolidine-1-propyne ligands. The R/S-Cu_14_ did not show emission in CH_2_Cl_2_, and was free of CPL. In CH_2_Cl_2_/n-hexane mixed solvents, the CuNCs showed AIE properties with red emission and CPL with *g_lum_* of 0.01 [29]. Although much has been achieved in the research of NCs-based CPL-active materials, two problems need to be further addressed: the fact that *g_lum_* is always low in solution and the fact that most CPL-active materials are built with chiral NCs instead of achiral NCs [30,31,32].

In this paper, we synthesized AuNCs modified by 4,6-diamino-2-mercaptopyrimidine hydrate (DPT-AuNCs) through an easy and mild method, and manipulated the co-assembly of DPT-AuNCs using organic acids, such as citric acid (CA), tartaric acid (TA), trimesic acid (Tra), phenylalanine (Phe) and lysine (Ly). On the one hand, it was found that CA could improve the emission of AuNCs with the formation of red emissive nanofibers (QY = 17.31%). At the same time, the luminescent nanofibers could selectively detect I^−^ with a limit of detection (LOD) of 2.75 μM. On the other hand, when DPT-AuNCs co-assemble with enantiomers of TA, circular dichroism (CD) signals could transfer into the achiral AuNCs and obtain CPL-active nanofibers with *g_lum_* of 9.4 × 10^−3^ and −4.5 × 10^−3^. Furthermore, in the ternary TA/CA/DPT-AuNCs complex system, the CPLs were amplified and given a very high *g_lum_* of up to 0.0436, exhibiting a 4.6-fold improvement. Herein, we not only significantly improved the emission of DPT-AuNCs, but also provided a convenient method for building CPL-active materials through straightforward achiral transfer, which could open a new avenue for construction of NC-based functional materials.

## 2. Materials and Methods

**Materials.** All the chemicals and solvents were analytically pure and used as received without processing. Aqueous solutions in all experiments were prepared using deionized water. Hydrogen tetrachloroaurate trihydrate (HAuCl_4_·3H_2_O) was produced by Sinopharm Chemical Reagent Co. (Shanghai, China). 4,6-Diamino-2-mercaptopyrimidine hydrate (DPT), citric acid (CA), phenylalanine (Phe), lysine (Ly), trimesic acid (Tra) and guanidineacetic acid (GA) were purchased from Macklin Biochemical Co., Ltd. (Shanghai, China). D-tartaric acid (D-TA), L-tartaric acid (L-TA), arginine (Arg), histidine (His) and mandelic acid (MA) were purchased from Aladdin Biochemical Technology Co., Ltd. (Shanghai, China).

**Synthesis and co-assembly of DPT-AuNCs.** In a typical process, an equal volume of aqueous solution of HAuCl_4_ (24 mM, 1 mL) was added to the prepared DPT (70 mM, 1 mL) in a clean glass vial. The mixture was stirred sharply at room temperature for 5 min until the color changed to brown, and the mixed solution was allowed to react at 20 °C for 24 h [33,34]. The DPT-AuNCs were diluted with water to 1/5 of the original concentration. Then, L-TA (6 mM, 60 μL) was added to the diluted DPT-AuNCs (1 mL). The L-TA/DPT-AuNC co-assembly was carried out 24 h before the following measurements. Both the D-TA/DPT-AuNCs and CA/DPT-AuNCs were prepared with this method.

**Characterizations.** High-resolution transmission electron microscopy (HR-TEM) images were taken on a JEOL JEM-2100PLUS system (JEOL, Tokyo, Tapan). UV-vis spectra were obtained on a Hitachi UV-vis 4100 spectrophotometer. The photoluminescence spectra were recorded on a F-7000 spectrofluorometer (Hitachi, Tokyo, Japan) with a quartz cell. The photoluminescence lifetime was recorded on a FLS1000 spectrometer (Edinburgh, UK). Scanning electron microscopy (SEM) observations were carried out on a Sigma 500 (Zeiss, Oberkochen, Germany). Circular dichroism (CD) spectra were recorded on a Jasco J-810 CD spectropolarimeter in 0.1 cm quartz cells with a scanning rate of 100 nm min^−1^ (JASCO, Tokyo, Japan). CPL spectra were measured on JASCO CPL-300 spectrophotometers (JASCO, Tokyo, Japan). The size and zeta potential of the samples were obtained on a Malvern Zetasizer Nano ZS system (Marvin Panalytical, Malvern, UK).

## 3. Results

### 3.1. Co-Assembly-Induced PL Enhancement

**Figure 1:**
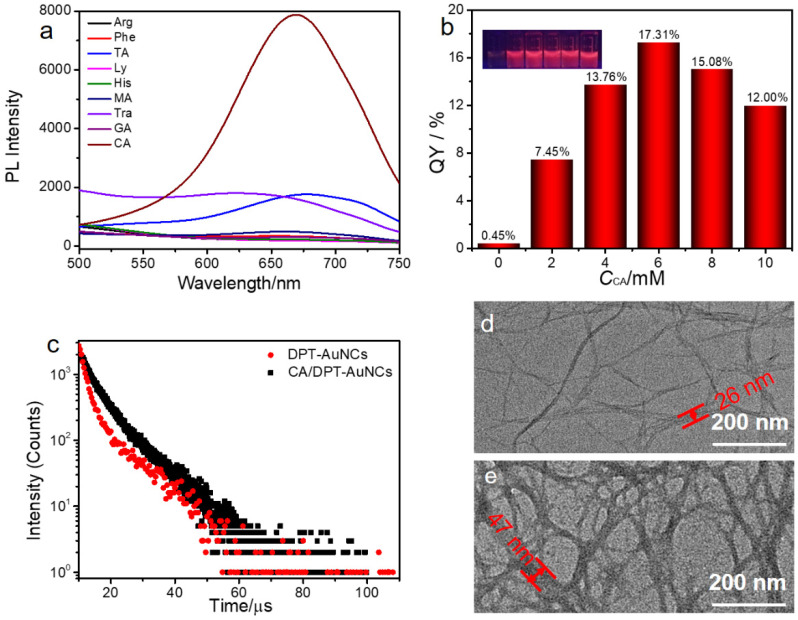
(**a**) PL spectra of DPT-AuNCs with different small molecules added (CA, TA, Tra, Phe, Ly, GA, Arg, His and MA, *c*_small molecules_ = 6 mM, λ_ex_ = 390 nm). (**b**) QY of DPT-AuNCs with different concentrations of CA (insets are photos of samples under handhold UV lamps with 365 nm). (**c**) PL decay curves of DPT-AuNCs and CA/DPT-AuNCs, *c*_CA_ = 6 mM. (**d**) TEM images of DPT-AuNCs. (**e**) TEM images of CA/DPT-AuNCs (*c*_CA_ = 6 mM).

DPT-AuNCs were synthesized according to the previously described method. The TEM and DLS results showed the successful preparation of nanosized DPT-AuNCs (Figures S1 and S2). The DPT-AuNC solution was pale yellow, emitted weak orange light under UV light at 365 nm and showed good solubility in water. The existence of amino and π-conjugate provided co-assembly sites for small molecules. Thus, we manipulated the co-assembly of DPT-AuNCs with various small molecules in efforts to improve the emission of DPT-AuNCs through an aggregation-induced emission (AIE) strategy. As shown in Figure 1a, the addition of some small molecules (Phe, Ly, GA, Arg, His and MA) only induced a slight PL enhancement, but the addition of some other small molecules (CA, TA and Tra) notably enhance the PL. CA, in particular, significantly enhanced the emission.

Thereafter, we studied the co-assembly behavior of DPT-AuNCs with CA (CA/DPT-AuNCs) in detail. Figure 1b and Figure S3 show that the PL intensity increased with increasing concentrations of CA (*c*_CA_) and reached its maximum at *c*_CA_ = 6 mM (λ_em_ = 670 nm). Further increasing the *c*_CA_ led to a slight decrease in the PL intensity. The emission enhancement was related to the interaction between CA and DPT-AuNCs. In our system, the contents of DPT-AuNCs were fixed, while with the increase in CA molecules (0~6 mM), the interaction between CA and DPT-AuNCs was enhanced, which enhanced emission. The emission reached the peak when *c*_CA_ = 6 mM. However, with excess additions of CA, the interaction between CA molecules might weaken the CA/DPT-AuNCs interaction, resulting in a slight weakening of the emission. This phenomenon is also reported for another co-assembly system [33] and for a good/poor mix solvents assembly system [35]. A strong luminescence enhancement (QY = 17.31%) was achieved after the addition of CA, compared with the weak emission of DPT-AuNCs alone (QY = 0.45%). As shown in Figure 1c, the CA/DPT-AuNCs showed a lifetime of 6.74 μs (χ^2^ = 1.0079) by integrating 3.21 μs (39.49%) and 9.04 μs (60.51%), which is longer than that of the primitive DPT-AuNCs (4.37 μs, χ^2^ = 1.0079) by the weighted sum of 1.85 μs (68.12%), and 9.77 μs (31.88%) (Figure 1c). The growth in lifetime is consistent with the enhancement of emission. Nanoarchitecture of the aggregates was studied by TEM. The formation of the DPT-AuNCs aggregates into slender nanofibers with a width of 26 ± 3 nm was observed (Figure 1d). With the addition of CA, the fiber nanostructures were also observed, while the width increased to 47 ± 3 nm (Figure 1e and Figure S4). The reason for the enhancement of the emission was explored. As shown in Figure S5, after the addition of CA to the DPT-AuNCs, the absorption of the sample was enhanced. A new band at 367 nm occurred, which might be attributed to the plasmonic absorption as a result of the formation of compact aggregates induced by CA. The compact aggregates formed by DPT-AuNCs/CA gave rise to larger DPT-AuNCs, which resulted in the plasmonic absorption [12]. The compact aggregation of DPT-AuNCs could also be confirmed by TEM (Figure 1d,e). In addition to the occurrence of plasmon, the aggregation could also be an important factor in enhancing the luminescence, because it weakens the non-radiative transition channel through restriction of molecular motion.

Various measurements were taken to analyze the mechanism of emission of CA/DPT-AuNCs. Firstly, 5 M urea, usually used to destroy hydrogen bonding, was added to the CA/DPT-AuNC solution. The PL of the CA/DPT-AuNCs was not quenched, but rather enhanced slightly (Figure S6). This result indicates the co-assembly of DPT-AuNCs/CA is not induced by hydrogen bonding. Secondly, to study the effect of the acidity on the luminescence of DPT-AuNCs, we added HNO_3_ to the DPT-AuNCs solution, achieving a pH value equal to the CA/DPT-AuNCs complex, while the PL intensity of DPT-AuNCs/H^+^ was much lower than that of CA/DPT-AuNCs. This indicates that the acidity had little effect on the emission of DPT-AuNCs (Figure S7). Lastly, we studied the effects of different concentrations of CA/DPT-AuNCs on zeta potential, and found that the zeta potential was almost constant (Figure S8) during the process. It is therefore reasonable to propose that the electrostatic interaction had no influence on the co-assembly between CA and DPT-AuNCs. Considering all these results together with structures of the small molecules shown in Figure S9, we speculate that the *n*···π (O···benzene of ligand, amino···C = O) and π···π (C = O···benzene of ligand) interaction was the dominant factor in the enhanced emission of the CA/DPT-AuNCs. CA exhibits the most abundant *n*···π/π···π interaction among the studied molecules, inducing the formation of “network clusters” and enhancing the emission of DPT−AuNCs [36].

### 3.2. I^−^ Detection

**Figure 2:**
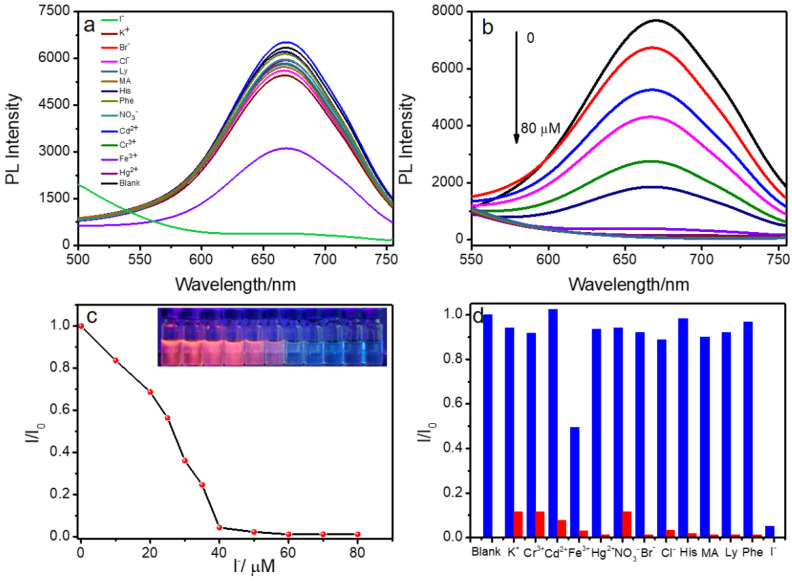
(**a**) PL spectra of CA/DPT-AuNCs with the addition of metal ions and small molecules (K^+^, Cr^3+^, Fe^3+^, Hg^2+^, Cd^2+^ NO_3_^−^, Br^−^, Cl^−^, I^−^, His, MA, Ly and Phe *c*_metal_ = 40 μM, *c*_small molecules_ = 40 μM). (**b**) Concentration-dependent PL spectra of DPT-AuNCs with different *c*_I−_. (**c**) PL intensity at 670 nm of CA/DPT-AuNCs upon addition of various quantities of I^−^. (**d**) Selectivity of CA/DPT-AuNCs toward I^−^: Blue bars represent the addition of various small molecules or metal ions to CA/DPT-AuNC solution, and red bars represent the subsequent addition of I^−^ (*c* = 40 μM) to the above solutions. Excitation wavelength is 390 nm.

The PL variation (enhancement or quenching) of probe materials could be used for detecting certain substances. Iodine (I) is one of the essential trace elements, and plays an important role in maintaining normal functions in the body [37,38,39]. Monitoring the level of I^−^ in the human body is considered an important part of maintaining human health. The detection of I^−^ with high selectivity and sensitivity is of great significance for tracing its physiological functions in organisms. Thus, considering the luminescent property of CA/DPT-AuNCs nanofibers, whose detection of small molecules and metal ions was explored, the changes in the luminescent intensity were monitored. As shown in Figure 2a, with the addition of 40 μM metal ions and small molecules, most molecules (K^+^, Cr^3+^, Fe^3+^, Hg^2+^, Cd^2+^ NO_3_^−^, Br^−^, Cl^−^, His, MA, Ly and Phe) presented slight changes in emission, except for I^−^, suggesting that the CA/DPT-AuNCs possessed a sensitivity toward I^−^. We studied the concentration-dependent PL spectra of DPT-AuNCs with regard to CA. As shown in Figure 2b, the PL intensity of CA/DPT-AuNCs decreased gradually with increasing concentrations of I^−^ (*c*_I−_). Moreover, the PL intensity and *c*_I−_ showed a linear relation ranging from 0 to 40 μM (Figure 2c). The limit of detection (LOD) was calculated to be 2.75 μM based on signal-to-noise ratio (S/N) of 3, which was comparable to that of some previous reports [40,41,42]. Simultaneously, the association constant (K) that could evaluate the interaction of DPT-AuNCs and CA can be calculated according to the Benesie–Hildebrand equation as follows:

(1) $$\frac{1}{I_{0}-I}=\frac{1}{I_{0}-I_{\max}}+\frac{1}{K\times \left(I_{0}-I\right)\times \left[I^{-}\right]}$$

where K is the association constant, I_0_ is the luminescent intensity of CA/DPT-AuNC solution, I is the luminescent intensity of CA/DPT-AuNCs containing different amounts of I^−^, and I_max_ is the maximum luminescence intensity of CA/DPT-AuNCs with I^−^. The 1/(I_0_ − I) value as a function of 1/[I^−^] showed a good linear dependence on the basis of luminescence intensity changes, and the K value between I^−^ and CA/DPT-AuNCs was calculated to be 1.04 × 10^5^ M^−1^ (Figure S10). The K value was very high, indicating I^−^ had strong interactions with the DPT-AuNCs. Additionally, high selectivity towards substance is an important indicator for evaluating the probe. Thus, the selectivity of CA/DPT-AuNCs toward I^−^ ions was also determined through competitive experiments. In Figure 2d, it can be observed that I^−^ induced strong quenching of the CA/DPT-AuNCs, even in the presence of other metal ions or small molecules, indicating CA/DPT-AuNCs are a promising luminescent probe specific for I^−^, and this high specificity toward I^−^ guarantees practical use in real samples.

**Figure 3:**
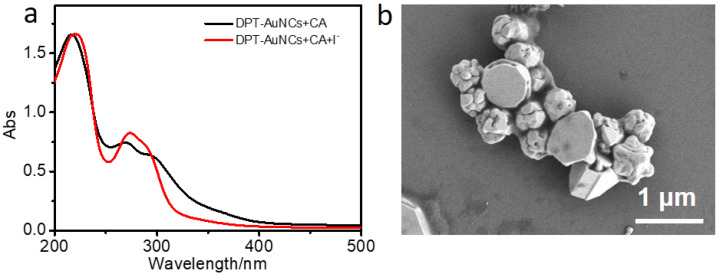
(**a**) UV-vis spectra of samples constituted by CA/DPT-AuNCs and CA/DPT-AuNCs/I^−^. (**b**) SEM image of CA/DPT-AuNCs/I^−^.

UV-vis spectra measurement was used to analyze the mechanism of I^−^ detection. As shown in Figure 3a, the absorption in the range of 300–400 nm decreased with the addition of I^−^. This range is located at the excitation scope of the DPT-AuNCs. Thus, with the decrease in absorption, the emission decreased. In SEM imagery, we found that the original slender nanofibers broke into a mixture of irregular particles, indicating that the I^−^ and the CA/DPT-AuNCs formed a new complex, causing the original structure to collapse.

### 3.3. Co-Assembly-Induced CPL Amplification

**Figure 4:**
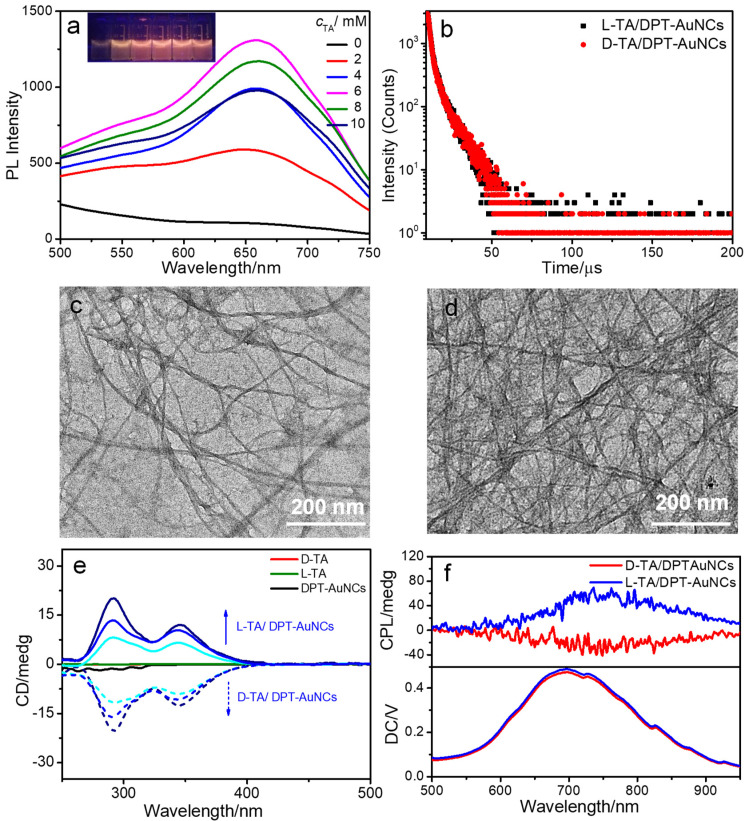
(**a**) QY of DPT-AuNCs with different concentrations of D-TA (insets are photos of samples under handhold UV lamps with 365 nm). (**b**) PL decay traces of L-TA/DPT-AuNCs and D-TA/DPT-AuNCs. (**c**,**d**) TEM image of L-TA/DPT-AuNCs and D-TA/DPT-AuNCs. (**e**) CD spectra of L-TA, D-TA, DPT-AuNCs, L-TA/DPT-AuNCs and D-TA/DPT-AuNCs complex solution (*c*_TA_ = 2 mM, 4 mM, 6 mM successively). (**f**) CPL spectra of the L/D-TA/DPT-AuNCs enantiomers in the solution state.

As shown in Figure 1a, chiral TA (D-TA) could also induce DPT-AuNC aggregates and improve the emission. Therefore, we explored the influence of different concentrations of D-TA on the intensity of DPT-AuNC emission. As shown in Figure 4a and Figure S11, PL intensity was improved with increasing *c_D_*_-TA_, and reached a peak at *c_D_*_-TA_ = 6 mM (λ_em_ = 656 nm). With further increases in the *c*_TA_, a slight decrease in the PL intensity was observed. The increase in lifetime was coincident with the enhanced emission of the DPT-AuNCs. The average phosphorescence lifetime of D-TA/DPT-AuNCs is 4.72 μs (χ^2^ = 1.0195), obtained by integrating of 1.78 μs (60.57%) and 9.24 μs (39.43%). We also studied the co-assembly behavior of L-TA/DPT-AuNCs. The PL intensity reached a maximum with *c_L_*_-TA_ = 6 mM with an average lifetime of 4.55 μs (1.79 μs (59.21%) and 8.55 μs (40.79%) χ^2^ = 1.0289) (Figure S12, Figure S13 and Figure 4b). TEM was utilized to examine the morphological structures of the co-assemblies. As shown in Figure 4c,d, the TA/DPT-AuNCs aggregated into ultralong nanofibers with a width of 29 ± 2 nm.

We studied the optical activity of DPT-AuNCs during the co-assembly process owing to the chirality of TA in nature. As is shown in Figure 4e, the L-TA/DPT-AuNCs and D-TA/DPT-AuNCs exhibited mirror image CD signals in the range of 200–400 nm, whereas the L/D-TA and DPT-AuNCs remained CD silent. Two positive and negative signals were observed at 292 nm and 346 nm for the L-TA/DPT-AuNCs and D-TA/DPT-AuNCs, respectively. With increasing *c*_TA_, the CD signals increased. It could be concluded that the chirality was transferred and amplified to nanofibers through the co-assembly process. Based on the excellent emission and optical activity of the nanofibers, we investigated the CPL property of the nanofibers to study their chirality in the excited state. As shown in Figure 4f, positive and negative CPL signals were observed for the L-TA/DPT-AuNCs and D-TA/DPT-AuNCs, respectively. The CPL response of L/D-TA/DPT-AuNCs solution occurred in the same wavelength region as their PL peak, centered at 750 nm with *g_lum_* of 9.4 × 10^−3^ and −4.5 × 10^−3^.

**Figure 5:**
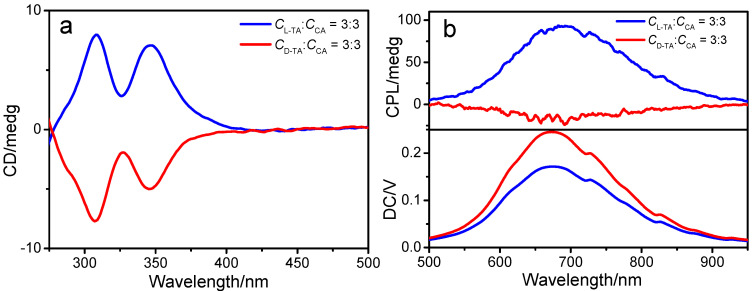
(**a**) CD spectra and (**b**) CPL spectra of L-TA/CA/DPT-AuNCs and D-TA/CA/DPT-AuNCs (*c*_TA_ = 3 mM; *c*_CA_ = 3 mM).

CPL-active materials with low *g_lum_* are limited in practical application. To achieve samples with higher *g_lum_*, ternary co-assembly of CA/TA/DPT-AuNCs was performed, based on the strong luminescent property of CA/DPT-AuNCs and the similar molecular structures of TA and CA. During the process, CD and CPL spectra were measured. Both the CD and CPL signals were positive. Firstly, with the addition of TA, CD signals were also observed at 250–400 nm in the case of CA/TA/DPT-AuNCs. Secondly, the CPL *g_lum_* of L-TA/CA/AuNCs and D-TA/CA/AuNCs mixture increased to 0.0436 and −0.006 (Figure 5b) with 4.6-fold and 1.3-fold enlargements, respectively, compared with the binary TA/DPT-AuNCs. Thus, this co-assembly system not only built the CPL-active materials through achiral AuNCs, but also further amplified the CPL signals.

## 4. Conclusions

In summary, supramolecular nanofibers with enhanced emission were obtained by the co-assembly of DPT-AuNCs with CA and chiral TA. It was confirmed that the *n*···π/π···π interaction plays a key role in the emission of DPT-AuNCs. Furthermore, the potential applications of the resultant luminescent nanofibers were explored. On one hand, the CA/DPT-AuNCs can be used as a superior sensor for I^−^ detection in water. On the other hand, the L/D-TA/DPT-AuNCs could generate new CPL properties. Notably, the *g_lum_* could be enhanced further in the CA/TA/DPT ternary co-assemblies, gaining potential as CPL materials. The proposed strategy for preparing luminescent and CPL-active materials from achiral AuNCs will significantly broaden the applications of metal clusters.

## Data Availability

The data are available on reasonable request from the corresponding author.

## Supplementary Materials

The supporting information can be downloaded at: https://www.mdpi.com/article/10.3390/nano12091453/s1. Figure S1: DLS result of DPT-AuNCs, Figure S2: HR-TEM image of DPT-AuNCs, Figure S3: (a) PL spectra and (b) PL intensity at the peak of emission of DPT-AuNCs with different concentration of CA, Figure S4: SEM image of DPT-AuNCs/CA, Figure S5: UV-vis spectra of DPT-AuNCs before and after addition of CA. Insert of the figure is the schematic representation of plasmon coupling of DPT-AuNCs before and after addition of CA, Figure S6: PL spectra of the CA/DPT-AuNC solution and CA/DPT-AuNCs induced by urea, Figure S7: PL spectra of the CA/DPT-AuNC and CA/DPT-AuNC/H+, Figure S8: Zeta potential of DPT-AuNCs with different concentrations of CA, Figure S9: structures of CA, TA and Tra molecules, Figure S10: The linear relationship of 1/(I-I0) vs. 1/I- for CA/DPT-AuNCs, Figure S11: (a) PL spectra and (b) PL intensity at the peak of emission of DPT-AuNCs with different concentration of D-TA, Figure S12: PL spectra of DPT-AuNCs with different concentrations of L-TA, Figure S13: (a) QY and (b) PL intensity at the peak of emission of DPT-AuNCs with different concentration of L-TA.
