# Supplementary material for: Manipulating the Assembly of Au Nanoclusters for Luminescence Enhancement and Circularly Polarized Luminescence
Source: Nanomaterials (Basel). 2022 Apr 25;12(9):1453. doi: 10.3390/nano12091453 (PMC9101361; doi:10.3390/nano12091453)
Supplement: Supplementary file 1 [file nanomaterials-12-01453-s001.zip › nanomaterials-1643416-supplementary.pdf]

nanomaterials

MDPI

Supplementary Materials

Manipulating the Assembly of Au Nanoclusters for Luminescence Enhancement and Circularly Polarized Luminescence

Chen Wang, Luyao Feng, Junxiao Liu, Jing Fu, Jinglin Shen * and Wei Qi *

School of Chemistry and Chemical Engineering, Qufu Normal University, Qufu 273165, China; wangchen@qfnu.edu.cn (C.W.); fengluyao@qfnu.edu.cn (L.F.); liujx@qfnu.edu.cn (J.L.); fujing@qfnu.edu.cn (J.F.)

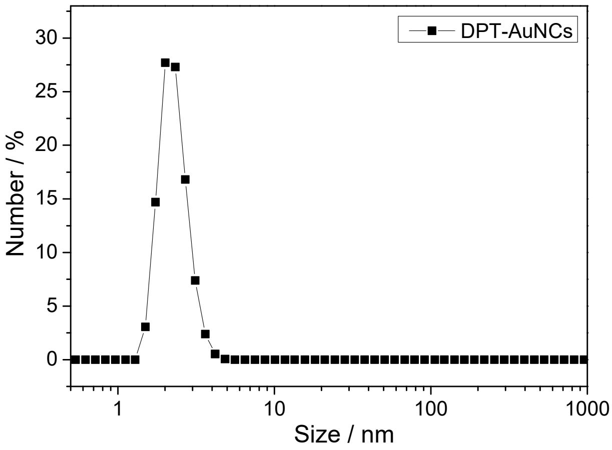

Figure S1. DLS result of DPT-AuNCs.

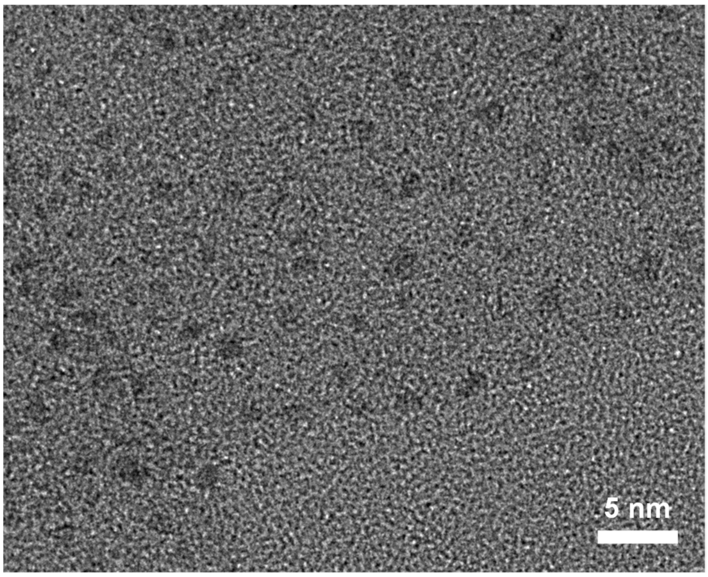

Figure S2. HR-TEM image of DPT-AuNCs.

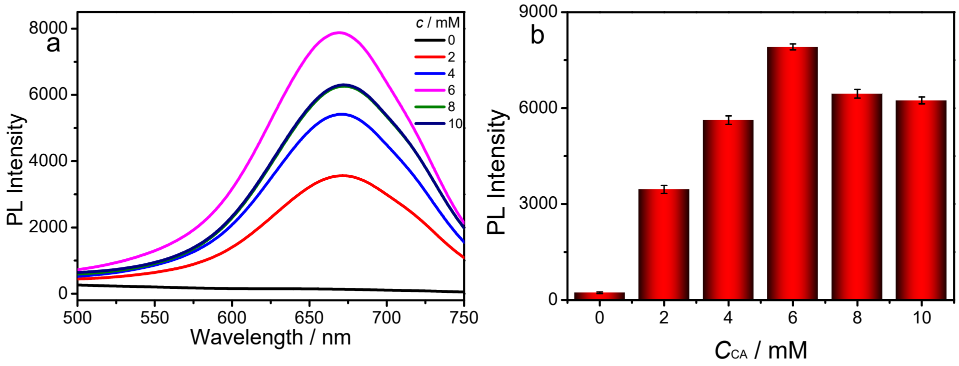

Figure S3. (**a**) PL spectra and (**b**) PL intensity at the peak of emission of DPT-AuNCs with different concentrations of CA.

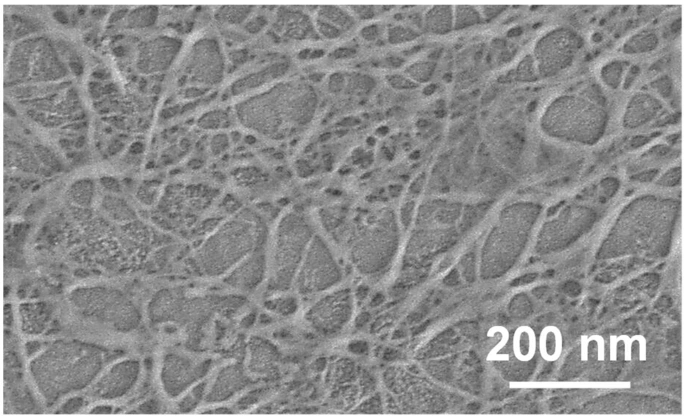

Figure S4. SEM image of DPT-AuNCs/CA.

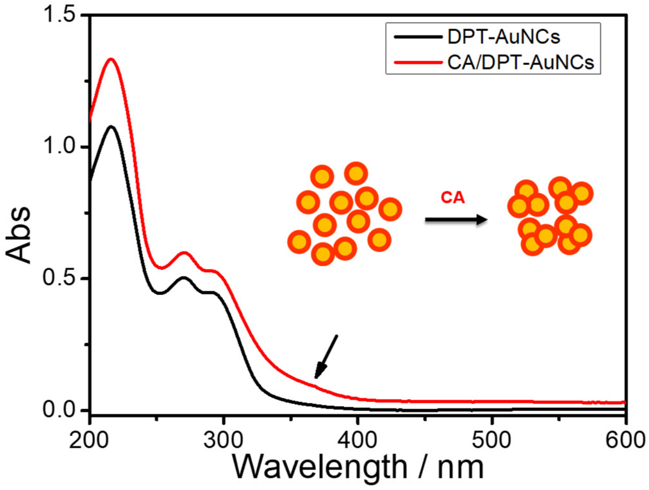

Figure S5. UV-vis spectra of DPT-AuNCs before and after addition of CA. The insert in the figure is a schematic representation of plasmon coupling of DPT-AuNCs before and after the addition of CA.

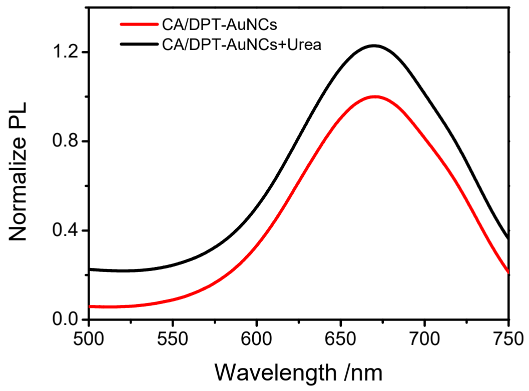

Figure S6. PL spectra of the CA/DPT-AuNC solution and CA/DPT-AuNCs induced by urea.

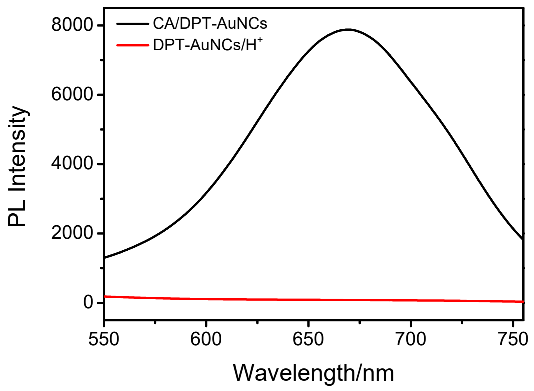

Figure S7. PL spectra of the CA / DPT-AuNC and CA / DPT-AuNC / H^{+}.

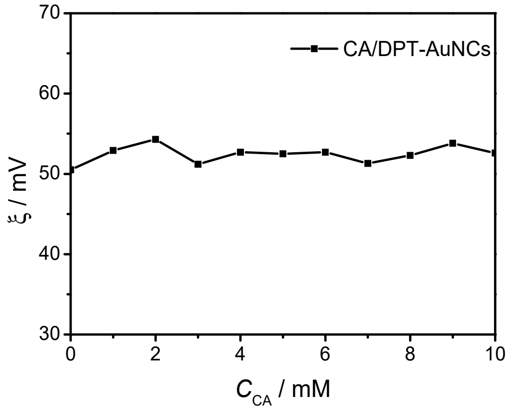

Figure S8. Zeta potential of DPT-AuNCs with different concentrations of CA.

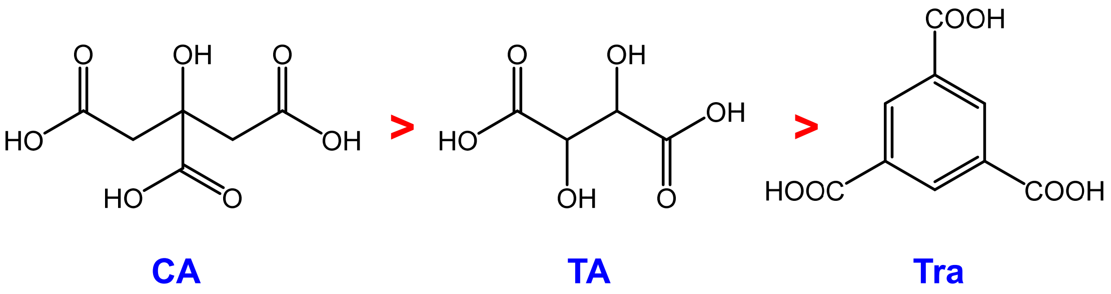

Figure S9. Structures of CA, TA and Tra molecules.

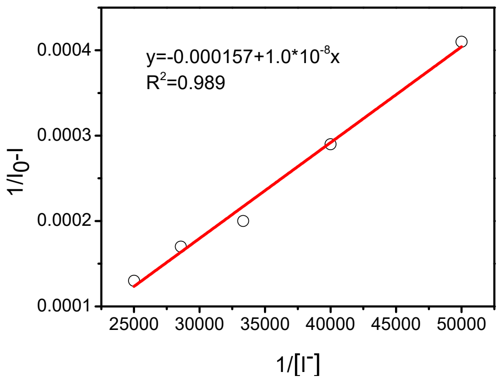

Figure S10. The linear relationship of 1 / (I-I$_0$) vs. 1 / I$^-$ for CA / DPT-AuNCs.

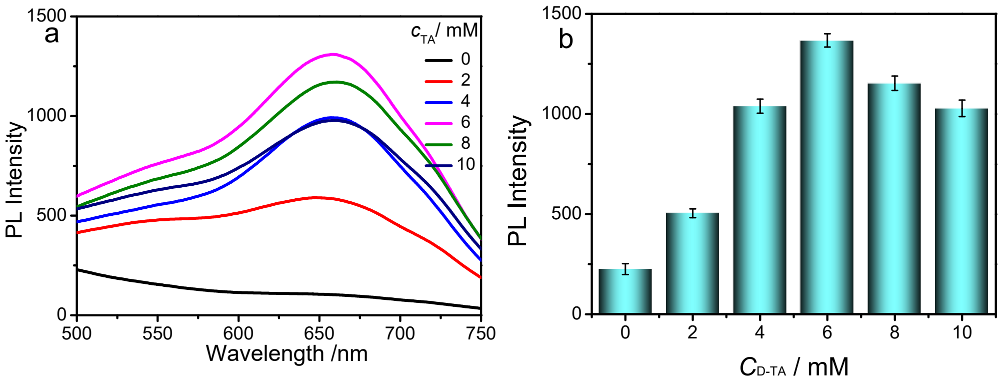

Figure S11. (**a**) PL spectra and (**b**) PL intensity at the peak of emission of DPT-AuNCs with different concentrations of D-TA.

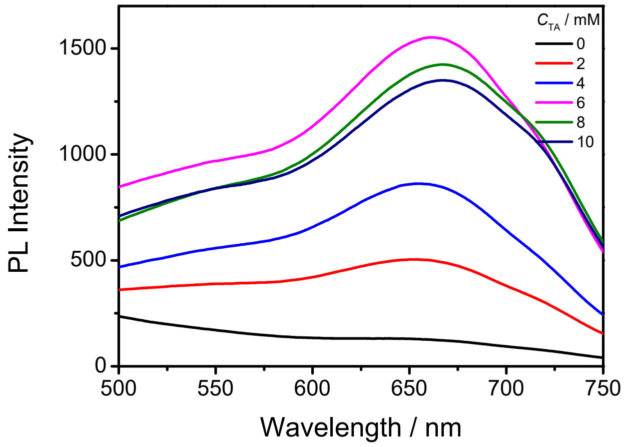

Figure S12. PL spectra of DPT-AuNCs with different concentrations of L-TA.

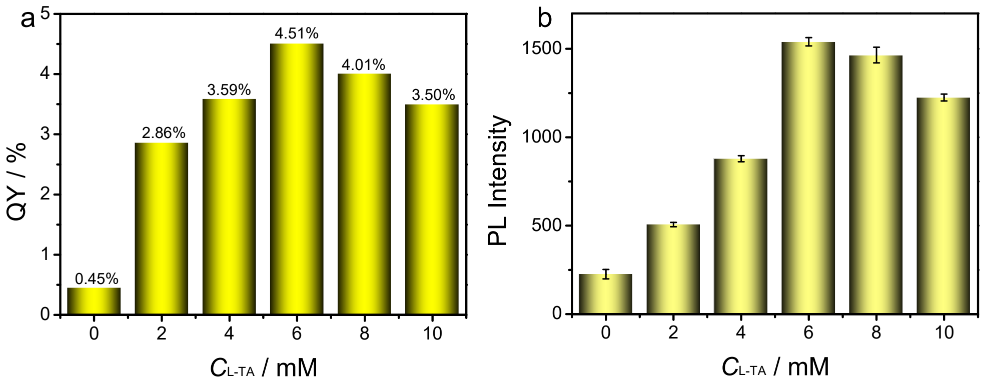

Figure S13. (**a**) QY and (**b**) PL intensity at the peak of emission of DPT-AuNCs with different concentrations of L-TA.
